# Classifying Oligometastatic Non-Small Cell Lung Cancer

**DOI:** 10.3390/cancers13194822

**Published:** 2021-09-27

**Authors:** Alisa N. Blumenthaler, Mara B. Antonoff

**Affiliations:** 1 Department of General Surgery, Indiana University School of Medicine, Indianapolis, IN 46202, USA; anblumen@iu.edu; 2 Department of Cardiovascular and Thoracic Surgery, University of Texas MD Anderson Cancer Center, Houston, TX 77030, USA

**Keywords:** oligometastasis, lung cancer, local consolidative therapy

## Abstract

**Simple Summary:**

Around half of patients with non-small cell lung cancer (NSCLC) have metastatic disease at the time of diagnosis. Some patients will have a limited number of metastatic sites, termed oligometastasis, rather than widespread disease. Aggressive treatment of oligometastasis has been supported by improved survival rates in retrospective studies, meta-analyses, and randomized phase II trials. Patient selection for aggressive local treatment of oligometastatic NSCLC would be facilitated by a common definition of what constitutes oligometastasis. We review the definitions of oligometastatic NSCLC proposed by consensus statements and those used in current treatment guidelines and previous trials of local consolidative therapy.

**Abstract:**

An oligometastatic cancer state was first postulated in the 1990s by Hellman and Weichselbaum and described limited metastatic spread to a single or few sites of disease. It was hypothesized that this metastatic entity falls along a continuum of the natural history of cancer progression from a localized primary tumor to widespread metastases. Support for oligometastatic non-small cell lung cancer (NSCLC) has since been provided by multiple retrospective studies and then prospective randomized trials demonstrating better survival in this patient population after aggressive consolidative treatment. However, the lack of a universal definition of oligometastatic NSCLC has hindered a comparison between different studies and prevented well-defined recommendations for local consolidative treatment in this patient population. Attempts have been made to establish a common definition for use in clinical management and for the identification of inclusion criteria for future trials. In this review, we seek to summarize the current definitions of oligometastatic NSCLC based on recent expert consensus statements, previous randomized trials, and current treatment guidelines and to highlight the continued variability in current practice.

## 1. Introduction

The concept of oligometastatic cancer was first postulated by Hellman and Weichselbaum in 1995, as they described an intermediate state of neoplastic malignant potential with limited metastatic spread and potential, existing on a continuum ranging from localized to widely disseminated disease states [1]. It was hypothesized that patients with oligometastatic disease could achieve long-term survival benefits if all sites of disease could be treated aggressively prior to progression of the metastatic malignant potential of the primary tumor or metastatic sites. Evidence for an oligometastatic state in non-small cell lung cancer (NSCLC) has since been provided, first by multiple retrospective studies, small phase II trials, and systematic reviews and more recently with randomized phase II trials demonstrating improved progression-free survival (PFS) and overall survival (OS) in patients who received locally aggressive treatment to all sites of disease [2,3,4,5,6,7]. However, the inclusion criteria for patients with oligometastatic NSCLC have varied widely amongst studies, which renders a comparison of the results challenging. It is imperative that a common definition of oligometastatic NSCLC be established for inclusion in future clinical trials in order to optimize the use of resources as well as patients’ outcomes. In this review, we hope to summarize the current definitions of synchronous oligometastatic NSCLC as presented by multiple national cancer guidelines and consensus working groups, as well as to discriminate oligometastatic disease from the distinct entity of oligoprogressive NSCLC.

## 2. Oligometastatic Disease

**Figure 1:**
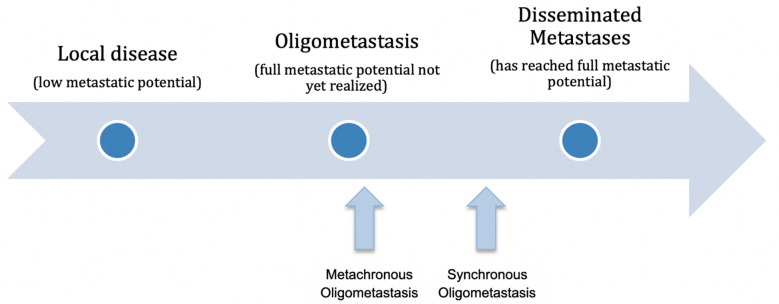
Oligometastasis represents an intermediate disease state along a continuum of malignant potential of the primary tumor and its metastases, as previously described by Hellman and Weichselbaum [1,11].

Close to 50% of patients with NSCLC have metastatic disease at the time of diagnosis, with an estimated 5-year survival rate of 6% [8,9]. However, a proportion of patients presenting with metastatic disease will have a limited metastatic burden, rather than widely disseminated disease. Oligometastasis can be broadly defined as a state of limited metastatic tumor burden, with only one or a few sites of metastasis and the potential to benefit from curative intent treatment [1]. Hellman and Weichselbaum theorized that the oligometastatic state is an intermediate state along a tumor’s natural historical progression from a localized primary tumor to a widely disseminated metastatic disease, which represents a condition in which the tumor’s full metastatic potential has not yet been realized (Figure 1). Early in the malignant trajectory, the metastatic cells from the primary tumor have a restricted capacity for growth in distant sites [10]. As the primary tumor grows and gains genetic mutations, cellular clones develop additional growth characteristics, the requirements for metastatic growth are less, and additional metastatic sites are established [10]. It was proposed that if eradication of the primary tumor and the oligometastatic sites could be achieved early in the metastatic continuum, progression to the widely disseminated state could be prevented, and cure potentially possible [1,11,12,13].

Since Hellman and Weichselbaum’s description, evidence for the presence of an oligometastatic state in NSCLC has been substantiated by a breadth of oncologic research. Initial support for improved outcomes after local treatment of oligometastases in NSCLC was identified in patients with solitary brain or adrenal metastases [14,15,16,17,18]. Additional proof of improved survival outcomes has emerged from retrospective analyses, meta-analyses, and single-arm prospective trials of local consolidative therapy (LCT) in patients with lesions in other (non-brain/non-adrenal) organs and even in those with more than a single metastasis [5,19,20,21,22,23,24,25,26]. More recently, multiple randomized phase II trials have provided additional, high-quality data in support of LCT as an attractive treatment strategy for oligometastatic NSCLC [2,3,27]. As lung cancer screening has improved and become more standardized and imaging modalities have become more sensitive, oligometastatic states have been more readily and confidently identified, with the most common sites of oligometastasis being the brain, lung, and adrenal gland, followed the bony skeleton [5,28,29,30]. Given the preponderance of evidence demonstrating significantly longer survival for patients after treatment of oligometastases compared to those with widely disseminated disease, the 8th Edition of the TNM staging classification included adjustments to the M stage subclassifications in order to account for these distinct metastatic entities [31].

Recently, the European Organization for Research and Treatment of Cancer (EORTC) and the European Society for Radiotherapy and Oncology (ESTRO) OligoCare Project undertook the task of defining biologically distinct oligometastatic disease states for all primary tumor types. The individual oligometastatic disease entities were classified based on the timing of oligometastasis diagnosis in relation to the diagnosis of the primary tumor and ongoing systemic therapy, a history of treated oligometastatic or polymetastatic disease, and the nature of the disease response to previous or ongoing treatment [32]. Broadly speaking, the umbrella term “oligometastasis” represents a state with “few” metastases. For the purposes of this review, our focus is on the disease entity which falls into the categories that the EORTC-ESTRO consensus refers to as “genuine oligometastasis” (no history of polymetastasis) and “de novo oligometastasis” (no history of previously treated oligometastatic disease) [32].

It has been difficult to specifically define oligometastatic disease in NSCLC, and different authors have employed widely variable definitions, in regard to both the number of metastatic lesions and the number of organs involved. Variability also exists regarding the inclusion of patients with metastases in specific sites, such as intracranial lesions or mediastinal nodal disease [33]. The current TNM staging paradigm characterizes M1a disease by the presence of pleural metastasis or contralateral pulmonary metastases in the absence of extrathoracic metastasis. M1b disease is classified as a single extrathoracic metastasis, while M1c subclassification includes multiple extrathoracic metastases, in one or more organs [31]. The current literature, however, does not directly align with this classification of oligometastasis, as a systematic review found that the maximum number of metastases for inclusion in studies of oligometastasis has ranged from one to eight sites, and up to three organs [33]. There is also variability in the definition of oligometastatic disease for the purpose of inclusion in previously published randomized phase II trials of LCT for oligometastatic NSCLC (Table 1). Many trials have not specified a maximum number of organs with metastasis allowable for study inclusion and have not included details about the inclusion of intrathoracic nodal disease as an independent metastatic site.

Even national treatment guidelines for NSCLC have failed to provide specific definitions for oligometastasis (Table 1). The National Comprehensive Cancer Network (NCCN) treatment guidelines do not specifically define what is referred to as “limited” metastatic disease for which LCT for oligometastasis can be considered, though it is noted in the guidelines that prior clinical trials have included limitations of three to five metastases [34]. The 2018 European Society for Medical Oncology (ESMO) guidelines recommend LCT for patients with less than three metastatic lesions, without specifying a limit of number of organs that can be included in the oligometastatic state [35]. This variability in the understanding of what constitutes an oligometastatic state has posed significant challenges when comparing outcomes across different trials, as well as when determining treatment paradigms for specific patient groups. Thus, attempts have been made to establish a single definition for oligometastatic NSCLC to better guide inclusion criteria for future clinical trials.

The European Organization of Research and Treatment of Cancer Lung Cancer Group (EORTC-LCG) recently undertook a systematic review, international survey, and multidisciplinary conference, with the goal of developing a consensus definition for oligometastatic NSCLC. A number of issues were taken under consideration in reaching agreement [36]. The overarching consensus was that oligometastatic disease can be considered to be present when there is the potential for modifying the disease course with the treatment of all disease sites by a technically feasible local treatment with an acceptable toxicity or risk profile [36]. The definition of treatment feasibility can be elusive in the era of consistently evolving and improving modalities for local therapy, such as minimally invasive surgical techniques, enhanced recovery surgical pathways, and more precise radiation therapy modalities (i.e., stereotactic body radiotherapy (SBRT), gated radiotherapy), which make LCT increasingly safe and well tolerated. This is an important point, as multiple studies have demonstrated the substantial benefit of a comprehensive treatment to all sites of disease. Patients who receive sub-comprehensive LCT, with treatment of one or more, but less than all, sites of disease, have been shown to experience worse survival outcomes than those who receive LCT to the primary tumor as well as to all sites of metastasis [4,7]. As we gain experience with offering LCT to wider patient populations and utilizing evolving treatment strategies, the potential for comprehensive LCT will also increase, and LCT be a valid treatment option for a growing number of patients.

Further, however, it is important to provide a more specific definition of the nature of the metastatic disease itself for patient inclusion in clinical trials as well as for risk stratification for those trials that include patients with oligometastatic, polymetastatic, and oligoprogressive disease. In considering the number of metastatic lesions that constitutes oligometastatic NSCLC, the EORTC-LCG was able to reach only a limited definition due to the sparsity of prospective data regarding the maximum number of lesions that can be beneficially treated with LCT [36]. The definition recommended by the EORTC-LCG based on disease burden includes a maximum of five metastatic lesions in up to three organs. Importantly, the presence of diffuse serosal metastases of the meninges, pericardium, pleura, or mesentery, as well as bone marrow involvement, were exclusionary for the EORTC-LCG definition of oligometastatic disease, as it was a general consensus in the working group that these sites are rarely, if ever, amenable to complete consolidation [36].

Pulmonary metastases are included in the maximum lesion count as defined by the EORTC-LCG. While nodules within the same lobe (T3) or lung (T4) are not considered metastases under the TNM definition, their presence may influence the feasibility of achieving comprehensive LCT. Further, the extent of intrathoracic tumor burden is an independent prognostic factor in patients being treated with LCT for oligometastatic NSCLC [4,5,37]. Similarly, the presence of intrathoracic nodal metastases is associated with a worse prognosis and decreased benefit from LCT in the case of oligometastasis [3,4,5,30]. The landmark randomized controlled trial by Gomez et al., which demonstrated improved PFS in patients randomized to LCT compared to those who received maintenance therapy or observation, included positive intrathoracic nodes in the total metastasis count for the purpose of inclusion in the trial, with intrathoracic nodes counting as a single site of disease (regardless of the total number of nodes involved) [2]. This same inclusion criterion has since been applied to current ongoing trials evaluating the role of LCT in oligo- and polymetastatic disease [38,39,40,41,42]. In contrast, mediastinal nodal disease has not been considered an independent metastatic site in the EORTC-LCG definition. While the EORTC consensus statement considers mediastinal nodes to be regional rather than metastatic disease, it does emphasize, however, that the extent of nodal disease can play a role in determining the feasibility of comprehensive LCT and thus is still important to consider [36].

## 3. Required Staging Workup 

A generally agreed upon requirement for oligometastatic disease is the confirmation that a patient’s cancer burden is indeed limited, rather than representative of an occult disseminated metastatic state [1,36]. Patients who experience disease recurrence after LCT more often experience systemic recurrence and appearance or progression of disease at sites different than those treated with LCT [4,12,23,27,43]. This fact underscores the importance of ruling out the presence of widespread polymetastatic disease to the extent of the capabilities and sensitivity of the current evaluation strategies. With continuously improving imaging modalities and the associated gains in sensitivity and specificity for identifying metastatic lesions, delineating an oligometastatic state may be confirmed with increasing confidence [29,44].

General agreement has been reached regarding the necessary staging workup that is required to identify an oligometastatic disease state. The EORTC-LCG consensus statement includes mandatory 18F-FDG PET/CT and imaging of the brain, of which MRI is the preferred modality [36]. The NCCN guidelines provide similar recommendations [34]. Mediastinal staging should be clinically inferred by PET/CT, with pathologic confirmation by endobronchial/endoscopic ultrasonography (EBUS/EUS) or mediastinoscopy, if the confirmation would influence treatment decision making. Pathologic confirmation of metastatic lesions, particularly in cases of suspected solitary metastasis, is recommended unless a multidisciplinary tumor board determines that the risk of biopsy outweighs any possible benefit or influence on clinical decision making [36]. For patients with a solitary metastasis in the liver or ipsilateral pleura, additional workup is recommended including liver MRI or thoracoscopy with biopsy, respectively.

## 4. Synchronous versus Metachronous Oligometastasis

The timing of metastasis may play a role in the prognostication of patients with oligometastatic NSCLC [5]. Synchronous oligometastases are those that are identified at the time of diagnosis of the primary tumor, while metachronous oligometastases are identified after treatment of the primary tumor [32,35,45]. The time between diagnosis of the primary tumor and that of a metachronous oligometastasis is poorly defined and is variable within the current literature [5,6,45]. However, the EORTC-ESTRO OligoCare consensus recommendation for classifying oligometastatic disease defined 6 months after the diagnosis of the primary tumor as the time frame to distinguish metachronous disease (greater than 6 months) from synchronous (6 months or less), as this time frame has been frequently used in the literature [32].

These two entities may represent different biological processes with differing prognoses after LCT. Synchronous oligometastases may represent a state further along the malignant metastatic continuum of disease, as the metastases are present at the time of diagnosis and may be more indicative of the presence of occult widespread, distant micrometastatic disease. On the other hand, metachronous oligometastases may be more representative of early metastatic potential, developing after the treatment of the primary tumor, with expansion of a limited number of cellular clones with metastatic potential [11,35] (Figure 1). In a large meta-analysis of single patient data, the diagnosis of metachronous oligometastasis was found to derive greater benefit from LCT in regard to OS, compared to synchronous oligometastasis [5]. However, this finding is not unanimous throughout the available literature, and further study is required. The majority of currently available prospective data supporting LCT for oligometastatic NSCLC are in the context of synchronous oligometastatic disease, and there are limited prospective data specific to metachronous oligometastatic NSCLC.

## 5. Oligoprogression

Oligoprogression differs from oligometastasis primarily by the timing of metastasis diagnosis or progression in relation to active systemic treatment [32]. Oligoprogression can occur in a “de novo” metachronous setting, with the identification of new oligometastatic lesions 6 months or more after primary tumor diagnosis, during active treatment, and in the absence of previously treated metastatic disease. Alternatively, Guckenberger et al also describe a “repeat oligoprogression” state, in which one or a few known oligometastases demonstrate progression during active systemic therapy, while all other known sites of disease remain stable or responsive to treatment [32]. This entity is typically considered in patients with NSCLC with genetic driver mutations. During systemic therapy, often with targeted therapy such as tyrosine kinase inhibitors (TKIs), many patients experience progression at a single site or only a few sites of disease, while the primary tumor and other metastatic sites remain stable or are even responsive to therapy [35,46]. It is believed that this oligoprogression at a single site is due to the expansion of a resistant clone at that site, while the rest of the disease remains susceptible to the targeted therapy [47]. These patients, like those with oligometastatic disease, may have the potential for long-term PFS when treated with LCT at the site of oligoprogression, with continuation of their systemic therapy [48,49,50,51].

## 6. Risk Stratification and Who Benefits Most from LCT

At this time, the completed prospective randomized trials of LCT for oligometastatic NSCLC have been stopped early due to significant improvement in primary endpoints of PFS in the LCT groups, compared to the groups receiving maintenance therapy (Table 2) [2,3,27]. While ending these trials early has signified enormous promise for this treatment paradigm, challenges have resulted. With the early termination of these trials, the ability to conduct subgroup analyses to identify factors associated with the greatest benefit from LCT has been limited. Given these limitations, some authors have attempted to utilize other means to identify groups of patients that stand to gain the most benefit from LCT.

In an individual patient data meta-analysis of 757 patients treated for oligometastatic NSCLC, Ashworth et al developed a risk stratification paradigm for patients with up to five metastases [5]. The authors suggest that patients with metachronous oligometastases without nodal disease constitute the “low-risk” group, with the greatest chance (up to 50% at 5 years) for long-term survival after LCT of their oligometastases and of the primary tumor. An “intermediate risk” group is defined by synchronous oligometastatic disease and N0 status. Patients in the “high-risk” group are those with synchronous oligometastases and positive nodal disease and have the lowest chance for durable benefit from LCT.

Another recent study by Mitchell et al. similarly attempted to identify, through subgroup analysis, patient characteristics that suggest the likelihood of benefit gained from LCT in synchronous oligometastasis. This study included 194 patients with synchronous oligometastatic NSCLC, identified through a natural language processing algorithm, including patients treated on the initial landmark trial by Gomez et al. as well as those treated off trial [2,4]. As shown in this study, 121 of these patients had LCT to all sites of disease (comprehensive LCT), 52 received LCT to some but not all sites of disease (sub-comprehensive LCT), and 21 did not undergo LCT. The greatest therapeutic benefit for LCT was seen in patients without intrathoracic nodal disease (lower intrathoracic stage), those without bony metastasis, those with non-squamous histology, and those with a lower number of metastatic sites [4].

The long-term results reported by Gomez et al. of a randomized phase II trial demonstrated non-significant decreases in survival benefit for patients with 2–3 metastases, a partial response to chemotherapy, N2/N3 disease, or CNS metastases [3]. Interestingly, the EGFR/EML4ALK status was associated with an improved OS, though this group of patients was small in this study. Results of another, small retrospective study in patients with metastatic EGFR-mutant NSCLC were suggestive of a PFS benefit after treatment with TKI in combination with LCT [52]. These findings form the basis for the initiation of a randomized trial evaluating LCT in this specific patient population, which is currently ongoing (Table 2) [39,40]. Other phase II trials have been unable to perform similar subgroup analyses due to limited sample sizes. The current ongoing phase III clinical trials in oligometastatic NSCLC will hopefully provide additional high-quality evidence regarding subgroups of patients that stand to gain the most benefit from LCT, in order to optimize patient selection for this aggressive-treatment algorithm.

## 7. Conclusions

Oligometastatic NSCLC represents a subset of patients with limited metastatic spread and the potential for achieving long-term survival, or even cure, with LCT to all sites of disease. The definition of oligometastasis by LCT treatment feasibility is vague and elusive, particularly in the context of ever-improving local treatment modalities. The EORTC-LCG consensus definition has provided a common understanding that can be a basis to establish inclusion criteria for future clinical trials, though even this definition is limited by the paucity of prospective data identifying the maximum number of lesions that stand to benefit from LCT. A complete staging workup is of utmost importance to confirm the absence of occult disseminated metastases and to further stratify patients according to prognosis and risk for recurrence and treatment benefit. Ongoing phase II and phase III trials will provide additional high-quality evidence for LCT of oligometastatic NSCLC and may shed light on patient factors that are most prognostic after an aggressive local therapy.

## Figures and Tables

**Table 1:** Comparison of Recent Definitions for Oligometastatic Non-Small Cell Lung Cancer in Clinical Trials, National Treatment Guidelines, and Consensus Definitions. Modern definitions for oligometastatic non-small cell lung cancer have varied widely.

Author	Year	Study Type	Maximum Number of Metastases	Maximum Number of Organ Sites	Maximum Lesions in Each Organ	Intrathoracic N+ as Metastasis	Pulmonary Lesion as Metastasis	Includes Intracranial Lesions	No Disease Progression after First Line Therapy	Notes
Ashworth [5]	2014	Meta-analysis	5	NS	NS	NS	Yes	Yes	NS	
Gomez [2,3]	2016, 2019	RCT phase II	3	NS	NS	Yes	NS	Yes	Yes	
Iyengar [27]	2018	RCT phase II	5	NS	3 in lung or liver	NS	Yes	Exclude uncontrolled intracranial	Yes	
Palma [12,13]	2019, 2020	RCT phase II	5	NS	3	NS	NS	Exclude if only site of disease	Yes	Not lung cancer-specific
Dingemans [36] (EORTC-LCG)	2019	Consensus working group	5	3	NS	No	Yes	Yes	NS	
TNM stage M1a [31]	2017	Staging Guidelines	1	1	1	No	Contralateral	Yes	NA	
NCCN [34]	2021	Treatment Guidelines	3-5	NS	NS	No	Treat as second primary	Yes	NS	
ESMO [35]	2018	Treatment Guidelines	3	NS	NS	NS	Treat as second primary	Yes	NS	

Abbreviations. NS, not specified; RCT, randomized controlled trial; EORTC-LCG, The European Organization of Research and Treatment of Cancer—Lung Cancer Group; NCCN, National Comprehensive Cancer Network; ESMO, European Society for Medical Oncology.

**Table 2:** Details of published and ongoing randomized trials of local consolidative therapy in patients with oligometastatic non-small cell lung cancer.

Study	Year	Trial Type	Number of Patients	Control Treatment	Intervention Treatment	Primary Endpoint	Reported Outcomes	Notes
Gomez [2,3]	2016	RCT Phase II	74 [49] ^b^	Maintenance treatment (can include observation)	LCT followed by maintenance treatment	PFS	Longer PFS in intervention group	
Iyengar [27]	2018	RCT Phase II	29	Maintenance chemotherapy alone	Stereotactic ablative radiotherapy (SAbR) + maintenance chemotherapy	PFS	Longer PFS in intervention group	
Northstar [39,42]	2018 ^a^	RCT Phase II	143 ^c^	Osimertinib alone	Osimertinib + surgery and/or radiation	PFS	Ongoing	EGFR-mutated cancers
Lonestar [41]	2017 ^a^	RCT Phase III	360 ^c^	Nivolumab and Ipilimumab	Nivolumab and ipilimumab + surgery and/or radiation	OS	Ongoing	

^a^ Year in which accrual began for the currently ongoing trials. ^b^ 74 patients were enrolled in the trial; 49 were randomized to the two treatment arms. ^c^ Anticipated accrual. Abbreviations. RCT, randomized controlled trial; LCT, local consolidative therapy; PFS, progression-free survival; OS, overall survival; EGFR, epidermal growth factor receptor.
